# Comprehensive study of the influence of reinforcements on the deformation mechanism and energy absorption performance of auxetic structure

**DOI:** 10.1038/s41598-026-53790-z

**Published:** 2026-05-19

**Authors:** Vítězslav Sobol, Ondřej Červinek, Jan Jaroš, Melanie Todt, Jakub Hurník, Daniel Koutný

**Affiliations:** 1 Faculty of Mechanical Engineering, Institute of Machine and Industrial Design, Brno University of Technology, Technická 2896/2, 616 69 Brno, Czech Republic; 2 Institute of Lightweight Design and Structural Biomechanics, Technische Universitaet Wien, Karlsplatz 13, 1040 Vienna, Austria

**Keywords:** Auxetic structure, Energy absorption, Reinforcements, Laser powder bed fusion (LPBF), Stainless steel 316L, NiTi alloy, Engineering, Materials science, Physics

## Abstract

The increasing speed of transportation requires outstanding performance of energy absorbers used in passive safety systems. These absorbers must deform at approximately constant force to safely dissipate kinetic energy during a collision. Structured materials exhibit a superior energy-absorption-to-weight ratio compared to bulk materials. Auxetic structures, with a negative Poisson’s ratio due to their unique internal geometry, further improve load redistribution and indentation resistance. However, their absorption capacity remains limited relative to conventional structures. Because no established method exists to systematically enhance the absorption capacity of auxetic structures, this study investigates the use of reinforcements and explains how they influence plastic hinge formation and the resulting energy dissipation mechanisms. Structures represented by reinforced re-entrant honeycomb are manufactured using Laser Powder Bed Fusion additive technology from stainless steel 316L and NiTi alloy. Complex experimental and numerical analyses show that reinforcements in re-entrant honeycomb improve absorption capacity mainly due to contact between deformed reinforcements and horizontal walls, rather than to the formation of new plastic hinges. By optimising the reinforcement placement, the unit cell can absorb three times more energy per unit mass than without reinforcements. Samples from NiTi alloy theoretically outperform those from stainless steel 316L in absorption capacity, but its practical applicability is constrained by LPBF processing challenges. Finally, a new Double Re-entrant Honeycomb structure is designed that can absorb more than 70% of energy per unit mass compared to the reference geometry, while decreasing engineering stress fluctuations by approximately 36%.

**Supplementary Information:**

The online version contains supplementary material available at 10.1038/s41598-026-53790-z.

## Introduction

Several factors determine the suitability of a structure for a high-energy absorption application. These factors are maximum force reaction, densification value, engineering (eng.) stress–strain characteristic, and the structure’s weight. The last three mentioned can be summarised by a parameter Specific Energy Absorption (*SEA*)^1,2^. Structured metamaterials (e.g. cellular structures) are considered better energy absorbers compared to a bulk material, because they can absorb the same amount of energy while reaching a much lower reaction force peak^3,4^. This means more efficient and safe energy dissipation^2,5–7^.

Auxetic structures are structures with a negative Poisson’s ratio caused by typical inner geometry, e.g. struts pointing inward the structure (re-entrant structures). Unlike a conventional structure (with a positive Poisson’s ratio), when tensile load is applied, the auxetic structure expands in the lateral direction; when compression is applied, it shrinks^8,9^. Auxetic structures offer better damping^10,11^, synclastic deformation^12–14^, or improved indentation resistance^15–17^, making them in demand in the aerospace, military, sports, and automotive industries for use in deformation zones of protective devices or bumpers^8^. However, the best energy absorbers (*SEA* > 20 J/g) are still structures with a positive Poisson’s ratio^2,18,19^. But in some applications, it can be valuable to combine the properties of auxetic structures with high-performance energy absorption^4,5,20^.

### Prerequisites of a high-performance energy absorber

To achieve the maximum value of *SEA* and an efficient use of the structure’s material, several prerequisites should be met.

First, the dissipated energy should be irreversible^1,3^. This is achievable using ductile material that can undergo high plastic deformation, and a deformation mechanism of rigid arm rotation around plastic hinges^3,4^. Such additively manufactured bulk material can be Stainless steel 316L^21^ or NiTi alloy^22,23^. Stainless steel 316L is a ductile material with a high elongation at break value (40%) and easy manufacturability, which makes it good for energy absorption^21^. NiTi alloy is a material with high potential for high-end engineering applications, such as aerospace and automotive^24^, including energy absorption, vibration reduction and structural adaptation^25^. The shape memory effect^25,26^ and ductility^22,23^ of the material can be beneficial for high-performance structures and reusable energy absorption^4^.

Second, an eng. stress–strain characteristic should be *plateau*, meaning that there are no peaks and valleys within the curve. Structures under compression have three main eng. stress–strain curve characteristics after elastic deformation: *softening* (high peak, then fast drop, then slow increase); *hardening* (stable increase of eng. stress) and *plateau* (stable constant eng. stress). These characteristics depend mainly on the structure’s internal geometry. While the *softening* and *hardening* characteristics have a model representation of that inner geometry, there is none for the plateau characteristic^27^. However, it can be achieved using structures with low relative density and a large number of individual cells^4,27^.

The third requirement is a high densification value. This means that the moment when all cell edges touch each other, resulting in steep increases in eng. stress, come in as late as possible^1,4,27^.

### High-performance structures

The two main groups of structures can be identified from comparative studies^1,2,28^. The first consists of structures with a high *SEA* value (> 20 J/g); the second consists of structures with an almost plateau characteristic of eng. stress–strain curve.

Structures in the first group were metal foams^18,29^, BCC-based designs^2,19,30^ or TPMS gyroid lattice^31^. However, the eng. stress–strain characteristic was strongly *hardening*, reaching only half of the efficiency of the *plateau* characteristic. On the other hand, structures in the second group were the exact opposite^28,32,33^—reaching only a fraction of the *SEA* value (< 3.5 J/g), while having good *plateau* characteristics. A common feature of these structures was the sinusoidal pattern of deformed arms and auxetic behaviour.

### Enhancing energy absorption capacity

When only part of the prerequisites for a high-performance energy absorber are met, several methods can be used to improve its performance. Gradient relative density can eliminate high peak stresses, enhance *SEA*, but also cause a strong *hardening* characteristic^19,34,35^. Arm thickness optimisation is useful for effective material use and for stabilizing the deformation pattern^36,37^. Reinforcements (e.g. extra ribs) can enhance the stiffness and *SEA* of the structure^38,39^, stabilise the deformation pattern^40^ and increase the number of plastic hinges^41^, but can also lead to a higher peak of eng. stress at the beginning of compression. However, this peak can be eliminated by using a slightly pre-deformed shape of struts in the as-built state^35,41^.

Mentioned studies investigated several different structures and methods of increasing *SEA*, but the effect of reinforcements on the deformation process was not studied in detail; the studies only quantitatively described the consequences of their use. Therefore, this study focuses on understanding how reinforcements influence deformation and, consequently, energy absorption capability of the Re-entrant Honeycomb (RHC) auxetic structure. RHC is used as a geometry with great potential for enhancing the absorption capacity using reinforcements^39^. By changing its horizontal arms, a modification called Double Re-entrant Honeycomb (DRHC) can be observed. This modification mimics the sinusoidal geometry presented by structures with good *plateau* characteristics^28,32,33^ and is also investigated in this study.

## Materials and methods

### Methodology

**Fig. 1:**
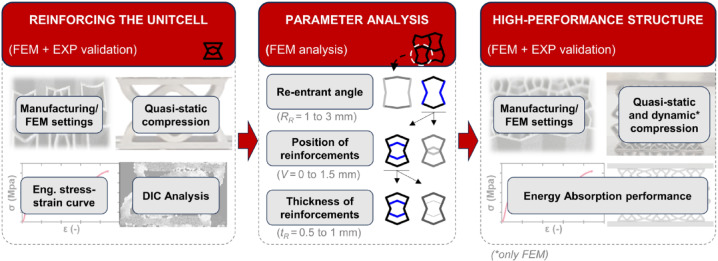
A scheme of the main workflow of the study.

The workflow is schematized in Fig. 1 and can be divided into three main phases. The first phase investigated the effect of reinforcements on deformation and energy absorption capacity both numerically and experimentally. Testing was done using a single unit cell with implemented reinforcements. By analysing this phase, knowledge about the deformation mechanism and optimal placement of the reinforcements was obtained. The second phase used these findings to narrow the range of the reinforcement’s placement in a single-parameter analysis to design new high-performance auxetic structures. In the third phase, the new designs were validated both numerically and experimentally by quasi-static compression tests and compared with a reference auxetic structure without reinforcements. The quasi-static test was used as a simple yet effective method to investigate the influence of geometric modifications on the deformation process. However, in the case of dynamic loading, the strain-hardening effect of the base material would be the dominant factor^42^, which was investigated in the last FEM analysis.

**Fig. 2:**
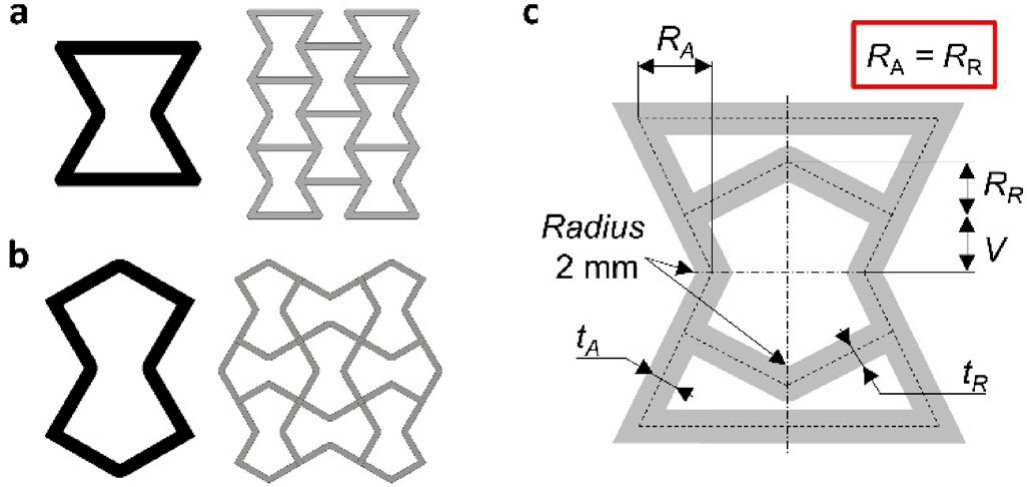
Two types of *re-entrant* auxetic structure: (**a**) RHC; (**b**) DRHC; and (**c**) tested parameters.

*Re-entrant honeycomb* (RHC, Fig. 2a) and *Double re-entrant honeycomb* (DRHC, Fig. 2b) were used in this paper. Only the DRHC type was considered for a high-performance structure.

An RHC cell with nominal dimensions 10 × 10 mm was used. To describe the influence of the reinforcement’s position (parameter $V$), the effect of the remaining parameters (deflection of arms ${R}_{A}$ and reinforcements ${R}_{R}$, thickness of arms ${t}_{A}$ and reinforcements ${t}_{R}$) had to be eliminated. For this reason, a reinforcement geometry was identical to that of the arms (${R}_{A}={R}_{R}=1.34 \mathrm{m}\mathrm{m}$ and ${t}_{A}={t}_{R}=1 \mathrm{m}\mathrm{m}$). Parameter $V$ ranged from 0 mm to 2.5 mm with a 0.5 mm step. The geometry and corresponding parameters are shown in Fig. 2c. Table 1 summarises the configurations investigated for a single unit cell.

**Table 1:** Investigated RHC unit cells and its names (*R*_*A*_ = 1.34 mm; *t*_*A*_ = *t*_*R*_ = 1.0 mm).

Config	No Reinf	V00	V05	V10	V15	V20	V25
*V* (mm)	–	0.0	0.5	1.0	1.5	2.0	2.5
Model							

### Materials used

Two types of materials were used: stainless steel 316L (EN 1.4404, TLS Technik GmbH, Germany) and NiTi alloy (CT-NiTi-EAAB, Carpenter Additive, United Kingdom). Mechanical properties such as density (${\rho}_{\mathrm{S}}$), Young’s modulus (${E}_{\mathrm{S}}$), Poisson’s ratio ($\mu$), Yield stress (${R}_{\mathrm{p}\mathrm{0,2}}$), and Ultimate tensile strength (${R}_{\mathrm{m}}$) are given in Table 2.

**Table 2:** Mechanical properties of stainless steel 316L^21^ and NiTi alloy^43^.

Material	ρ_S_ (kg m^−3^)	E_S_ (GPa)	µ (–)	R_p0,2_ (MPa)	R_m_ (MPa)
316L	7950	166	*0.25*	450	541
NiTi	6340	30.75	*0.3*	430	> 1514

The samples were manufactured using an SLM 280^HL^ machine (SLM Solutions Group AG, Lübeck, Germany) with the process parameters given in Table 3.

**Table 3:** Process parameters for LPBF manufacturing from^21^ and^44^.

Process parameter	316L	NiTi
Platform heating	100 °C	200 °C
Inert atmosphere	N_2_	Ar
Oxygen level	< 0.2%	< 0.2%
Layer thickness	50 µm	50 µm
Scanning contours	100 W, 300 mm/s	360 W, 1250 mm/s
Hatching	275 W, 700 mm/s	360 W, 1250 mm/s
Hatch distance	120 µm	115 µm
Fill contours	150 W, 400 mm/s	–

### FEM analysis

**Fig. 3:**
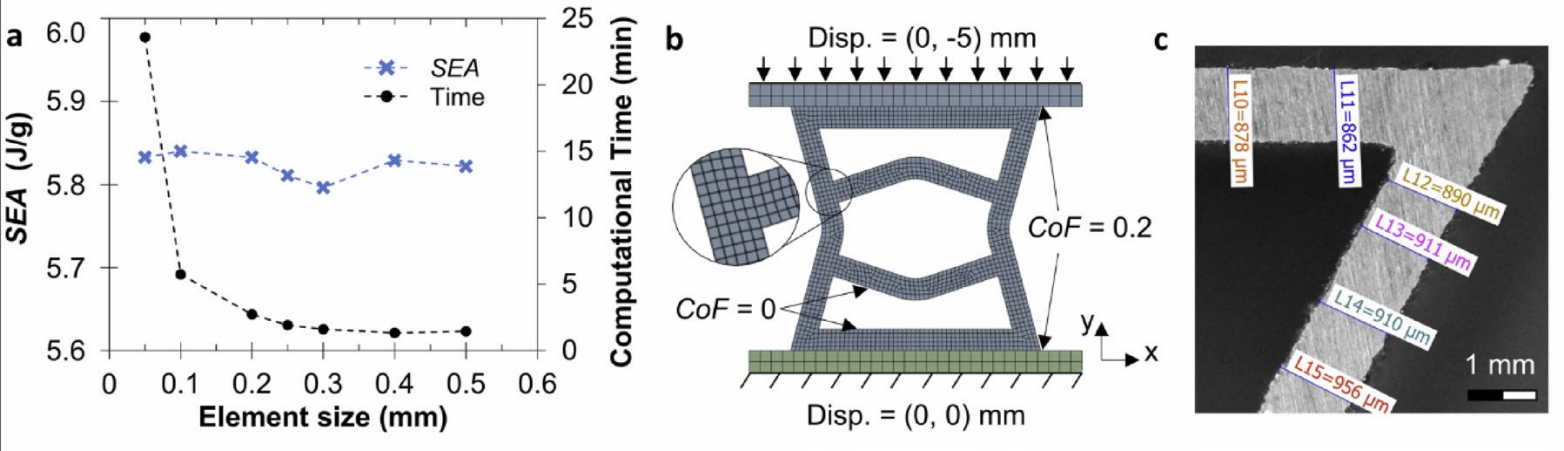
(**a**) Mesh independence analysis; (**b**) Boundary conditions and mesh for FEM simulations; (**c**) Real wall thickness measured on manufactured samples (detail).

FEM analysis was performed in ANSYS Workbench 2025 R1 (Ansys, Inc., Canonsburg, USA). The simulations were done in the Static Structural module as a non-linear problem. A 2D *Plane Strain* geometry was used. The wall thickness in the CAD model was individually adjusted to match the average measured value from the manufactured samples. A new bilinear material model for 316L was created using properties listed in Table 2. It was calibrated with experimental testing, so the tangent modulus was set to ${E}_{t}=890 \mathrm{M}\mathrm{P}\mathrm{a}$. For NiTi alloy, a multilinear material model was defined according to^43^. Both material models were considered without failure criteria. The mesh was generated using *Plane 183* elements (quadrilateral with 8 nodes). Based on the mesh independence analysis (Fig. 3a), the mesh size was set to 0.2 mm (> 5 elements across the thickness), balancing accuracy, computational time and sufficient resolution of strain maps.

Quasi-static compression tests were performed with boundary conditions assigned to a rigid upper and bottom plate (Fig. 3b). Contacts between arms and reinforcements were set as *Frictionless*, while contacts between the sample and plates were set as *Frictional* with a coefficient of friction of 0.2.

### Experimental validation

Each designed configuration was tested in 3 samples. Outer dimensions were checked after manufacturing using callipers. Wall thickness was measured using an Olympus SZX7 (Evident, Tokyo, Japan) at least 10 locations to determine the actual values of ${t}_{A}$ and ${t}_{R}$ (Fig. 3c).

The uniaxial quasi-static compression test was performed with the testing machine SHIMADZU AGX-100kNV2 (SHIMADZU CORPORATION, Japan). Compression speed was set to 1 mm/min up to a compression of 5 mm.

The compression tests were recorded using two DIC cameras (5MPx CMOS camera, 2 Hz; Istra 4D V4.10 software, Dantec Dynamics a/s, Skovlunde, Denmark).

### High-performance auxetic structure

A single-parameter analysis was used to achieve stable deformation and enhance the structure’s energy-absorption performance. The findings from the analysis of the single unit cell were used to narrow the range of the reinforcement’s placement for the new geometry of the structure with optimal values of ${R}_{A}, {R}_{R} \mathrm{a}\mathrm{n}\mathrm{d} {t}_{R}$. The simulation setup was similar to that described in section “FEM analysis”.

**Fig. 4:**
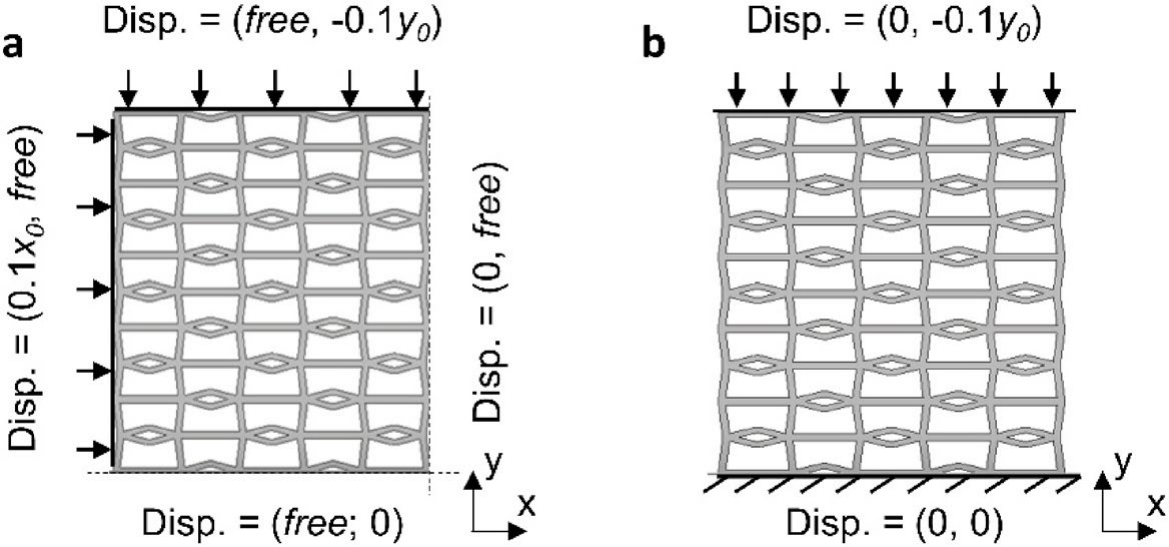
Boundary conditions for: (**a**) determination of the minimum number of cells; (**b**) single-parameter analysis (x_0_ and y_0_ are initial dimensions of the structure).

To test the structures validly, it was necessary to determine the minimum number of cells. A sufficient number of cells meant that their further increase did not change the *SEA* by more than 10%. Simulations were performed for DRHC structures (with implemented reinforcements) with an initial cell size of 1 × 1, 3 × 3, 5 × 5, 7 × 7, and 9 × 9. Boundary conditions were as follows: the right and bottom sides of the structure were prevented from moving in a direction perpendicular to these sides. The remaining outer edges of the structure were compressed by a value, causing 10% strain of the whole structure (Fig. 4a). This method eliminated the structure’s lateral distortion, so the *SEA* value was affected only by the number of cells.

The boundary conditions for the single-parameter analysis are shown in Fig. 4b. The range of investigated parameters was: ${R}_{A}={R}_{R}=1, 1.5, 2 \mathrm{a}\mathrm{n}\mathrm{d} 3 \mathrm{m}\mathrm{m}$; $V=$ 0, 1 and 1.5 mm; ${t}_{R}=$ 0.5, 0.75, 1 mm and *combined***.** The *combined* thickness differentiated between horizontal (${t}_{R}=$ 0.75 mm) and vertical (${t}_{R}=$ 0.5 mm) reinforcements. The goal was to achieve simultaneous deformation of all reinforcements, which was not observed in preliminary simulations with uniform thickness.

Manufacturing and testing of the structures were performed in the same manner as for the single unit cell (section “FEM analysis” and “Experimental validation”). Boundary conditions for numerical analysis were the same as in Fig. 3b, but with a compression of 30 mm. Compression speed for experimental testing was set to 5 mm/min up to a compression of 30 mm. Finally, an explicit dynamic analysis was performed to assess the structure’s deformation pattern under dynamic loading conditions. The impact velocities were 15 m/s and 55 m/s. The material model from^45^ was used.

The obtained force–displacement curves from all numerical and experimental analyses were transformed into eng. stress–strain curve according to $\sigma =F/{S}_{str}$, where $\sigma$ is eng. stress, $F$ is obtained reaction force, ${S}_{\mathrm{s}\mathrm{t}\mathrm{r}}$ is the area of specimen projection in the direction of the force; and $\varepsilon =y/{y}_{0}$, where $\varepsilon$ is eng. strain, $y$ is the actual compression value, and ${y}_{0}$ is the initial height of the specimen. Other output metrics were relative density (${\rho}_{\mathrm{R}\mathrm{E}\mathrm{L}}$), energy absorption efficiency ($\eta$), onset of densification (${\varepsilon}_{D}$), densification efficiency (${\eta}_{D}$), Specific Energy Absorption (*SEA*), deviation from plateau characteristic (${R}_{\sigma \_plateau}$) and plateau stress (${\sigma}_{plateau}$). Their descriptions are listed in the Supplementary Material.

## Results and discussion

The following section presents the results from experimental testing and FEM analysis, along with a discussion of their implications for the design of a high-performance structure for energy absorption.

### FEM analysis and experimental validation

A check of samples after manufacturing showed that the actual outer dimensions and wall thickness were, on average, (99.7 ± 0.5) % and (90.6 ± 3.2) % of the nominal CAD model, respectively. Measured wall thickness was used to adjust the CAD models for FEM analysis. The evaluated data from FEM and experimental analysis are summarised in the Supplementary Material.

#### Stainless steel 316L

**Fig. 5:**
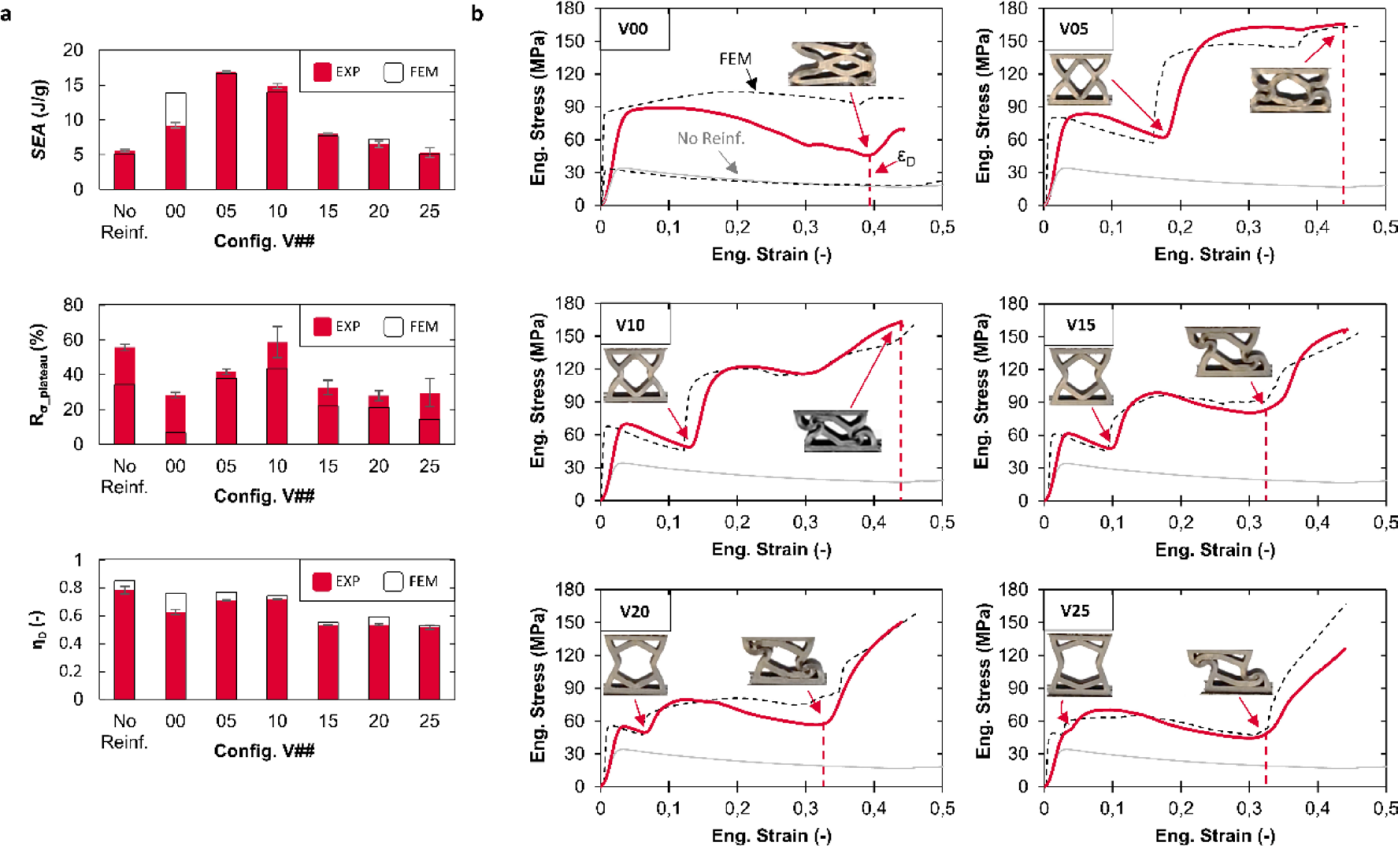
(**a**) Summary of the output metrics for unit cells from stainless steel 316L; (**b**) Eng. stress–strain curves with highlighted moments of reinforcements collision and densification; experimental data (red line) are compared with FEM (black dashed line) and with No Reinf. configuration (grey line for experimental data, black dashed line for FEM).

The comparison of the results (Fig. 5a) showed an increase in *SEA* when the reinforcements were placed closer to the centre of the cell (except V00). The increase in absorbed energy was correlated with a higher value of ${R}_{\sigma \_plateau}$. However, the ${R}_{\sigma \_plateau}$ value was mostly lower for reinforced unit cells than for No Reinf. configuration, so the reinforcements showed great potential to enhance energy absorption performance.

The inward deflection of the arms caused the deformation of the reinforcements. The V00 configuration was the exception, with reinforcements placed directly in the middle of the cell. This led to their merging and over-reinforcement of the region, resulting in lateral distortion. For this reason, no deformation of the reinforcements occurred, which made the σ(ε) dependence completely different compared to the remaining configurations (Fig. 5b).

**Fig. 6:**
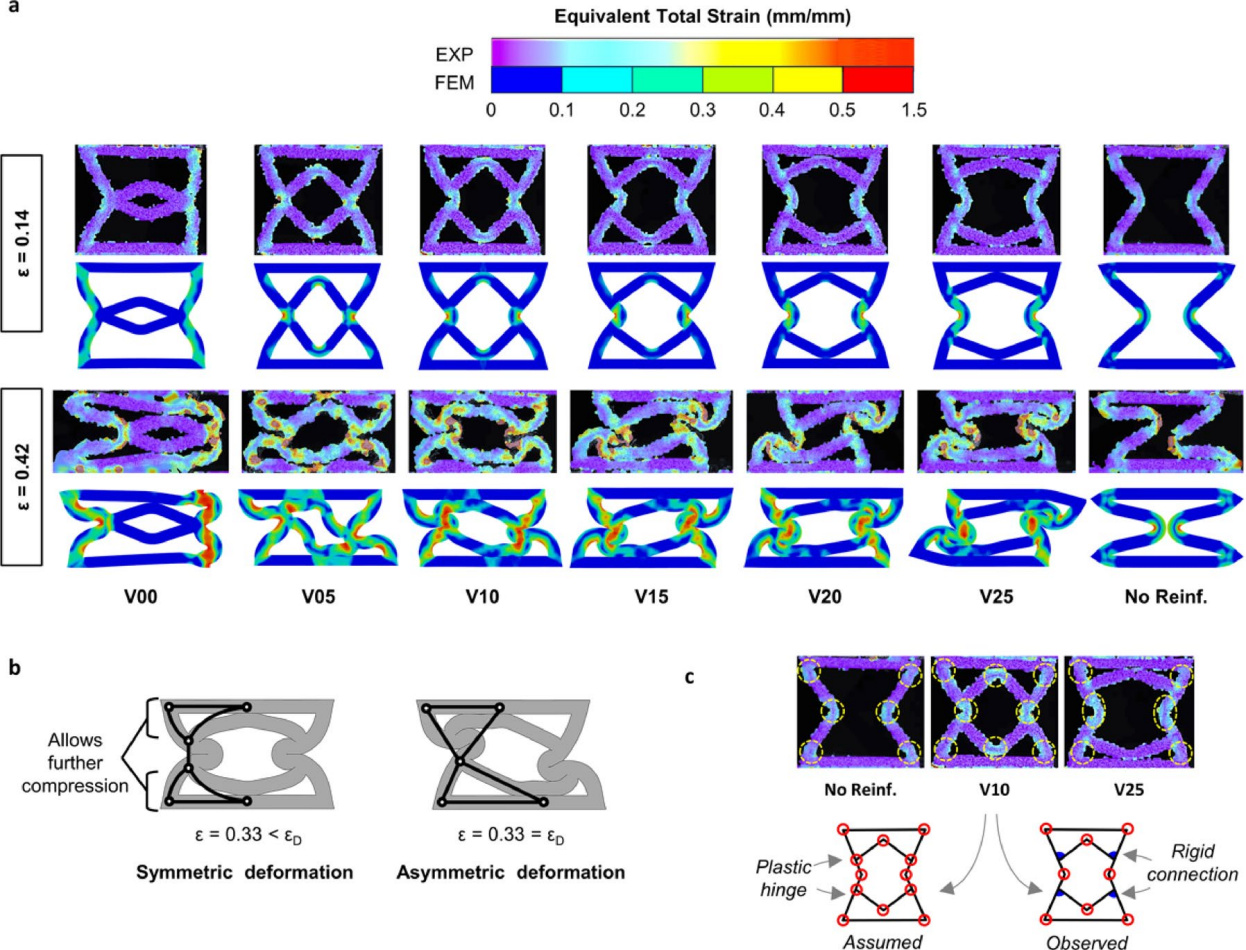
(**a**) Strain maps for unit cells from stainless steel 316L at ε = 0.14—main locations of initialisation of plastic hinges, and at ε = 0.42—main locations contributing to energy dissipation; (**b**) Effect of an a/symmetric deformation on wall layering and densification value; (**c**) Assumed and observed formation of plastic hinges.

There are two characteristic regions within the σ(ε) curve, each exhibiting a plateau-like behaviour. They are separated by the moment of contact between the deformed reinforcements and the horizontal arms. This contact occurred for all configurations, but it occurred later for the reinforcement closer to the centre. Therefore, the deformed reinforcements were oriented more vertically (in the loading direction). This led to a higher increase in the eng. stress and hence the *SEA* value. On the other hand, as the reinforcements were further from the centre, lower eng. stress peak was observed, which negatively impacted the total amount of absorbed energy. Reinforcements in configurations V15, V20, and V25 were subjected to minimal deformation (due to the proximity of the horizontal arms), and cell compression was primarily achieved through deformation of the arms in the central region (Fig. 6a).

It is also evident that the peak eng. stress within the first plateau region was up to three times higher for reinforced unit cells. The reinforcements prevented the arms from deflecting inward, thereby increasing the force required to compress the unit cell. The closer the reinforcements were to the centre of the cell, the greater resistance they offered to arm deflection due to the larger lever arm.

Given the increased relative density resulting from the implemented reinforcements, densification was expected to occur earlier, as described by^4^ and^39^. But according to the densification efficiency (${\eta}_{D}$), which accounts the presence of reinforcements, densification occurred too early compared with the specimen without reinforcements (${\eta}_{D \,V\#\#}\approx 0.57; {\eta}_{D\, No\, Reinf.}=0.78$). This was due to a slight asymmetric deformation after the contact between the reinforcements and the horizontal arms. It resulted in the layering of the deformed walls, which created a kind of frame structure, leading to earlier densification (Fig. 6b).

Strain maps in Fig. 6a highlight the plasticising regions where the energy was primarily dissipated. The first case, at ε = 0.14, captures the initiation zones where plastic hinges formed. These locations became the main points around which the “rigid” arms rotated. No significant areas of plasticised material were formed at the connections between the reinforcements and the arms. Compared to the No Reinf., new plastic hinges formed only at the inflexion point of the reinforcements (Fig. 6c), at least in configurations with reinforcements closer to the centre (V05, V10, V15). It was assumed according to^41^ that new hinges would form at the connections between the reinforcements and the arms, too. However, these connections behaved more like a rigid joint than a rotational one. These findings can guide the further design of novel structures by incorporating additional inflexion points in the reinforcements to increase the number of plastic hinges that dissipate energy.

The second case, at ε = 0.42, shows the state of the sample slightly before the complete compression. The engagement of the primarily formed plastic hinges in energy dissipation is clearly captured in the strain maps. For samples V05 and V10, the plasticised regions were approximately evenly distributed throughout the sample, i.e., including the reinforcements. For the remaining configurations, the reinforcements contributed less to energy dissipation. Because the reinforcements were further from the cell’s centre, this led to the creation of a low-stiffness area at the centre of the cell. Therefore, only the deformation of this part was responsible for most of the total compression, as reflected in the amount of deformed material and the dissipated energy.

The FEM simulation results were in very good agreement with the experiments, as shown in Figs. 5 and 6. The largest difference was obtained for the V00 configuration (all samples), where the lateral distortion was captured in a different mode. One sample of the No Reinf. configuration showed asymmetric deformation during experimental testing, unlike in FEM analysis, but this affected the eng. stress–strain curve only after $\varepsilon$ = 0.473. This shows a significant sensitivity of both configurations to minor geometric and loading imperfections (e.g., exact wall thickness, off-centre placement of the sample, non-parallel top and bottom surfaces, etc.).

#### NiTi alloy

**Fig. 7:**
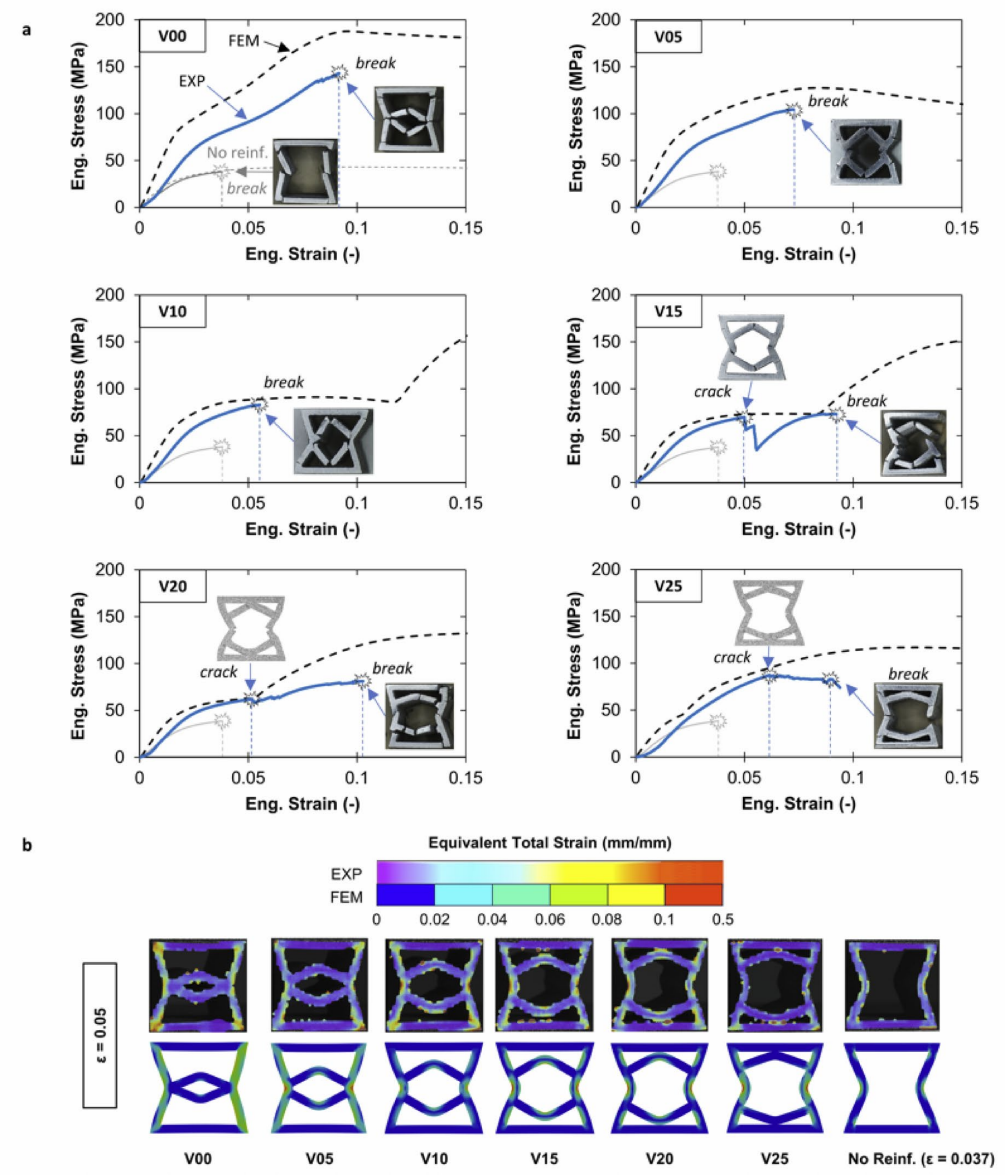
(**a**) Eng. stress–strain curves for NiTi unit cells with highlighted moments of first cracks (*crack*) and complete destruction (*break*); experimental data (blue line) is compared with FEM (black/grey dashed line) and with a cell without reinforcement (grey line). (**b**) Strain maps for unit cells from NiTi at ε = 0.05.

Similar results to those with stainless steel were assumed for the NiTi alloy, but experimental testing of single-unit cells revealed unexpected material behaviour. Fabricated NiTi alloy samples exhibited insufficient ductility of the material during compression tests. As can be seen in Fig. 7a*,* the single unit cells were able to reach an eng. strain of only about 10%.

Maximum stress and strain values within the bulk material were located, as expected, at the hinge positions—these can be visible as locations of material rupture in Fig. 7a and in strain maps in Fig. 7b. According to DIC records and FEM simulations, the maximum stress and strain were approximately 1250 MPa and 0.18 mm/mm, respectively. Compared to the material model definition^43^, it is beyond the super-elastic behaviour of the NiTi alloy, and the material should be in the Martensite phase. However, it is still below the Ultimate tensile strength. Possible causes of these poor mechanical properties include a thin-walled geometry and/or insufficient process parameters for manufacturing such a geometry. Because of the thin-walled geometry, the mechanical properties could be different from those of the bulk material. Thin walls usually have greater surface roughness and a much faster cooling rate, resulting in poor mechanical properties, increased stress concentrations, and lower ductility^46–48^. It can also be difficult to produce defect-free NiTi alloy as it is susceptible to cracking during manufacturing due to high residual stresses, nickel evaporation or incompletely melted powder^49^.

Within the configurations tested, two distinct events could be identified, labelled as a *crack* and a *break* in Fig. 7a. The *crack* label corresponds to crack formation in the hinges; however, further compression led to similar or even higher eng. stress. On the other hand, the *break* label corresponds with complete destruction of the sample or no further increase in eng. stress. The *break* event was observed in all configurations, but the *crack* occurred only in the V15, V20, and V25 configurations, i.e., configurations with reinforcements further from the cell’s centre. Such placement of the reinforcements resulted in a smaller gap between them and the horizontal arms, leading to an earlier collision during deformation. This contact stiffened the geometry, preventing the material from exceeding its load capacity in a sufficient volume to cause immediate destruction of the specimen. For the remaining configurations, i.e. No Reinf., V00, V05, and V10, the maximum stress and strain values in the formed hinges were far beyond the material limits, even before contact between the reinforcements and the horizontal arms. Therefore, only the *break* event was present. Eng. stress–strain curves obtained from FEM analysis followed the same trend as the experimental ones, until the fracture of the material.

**Fig. 8:**
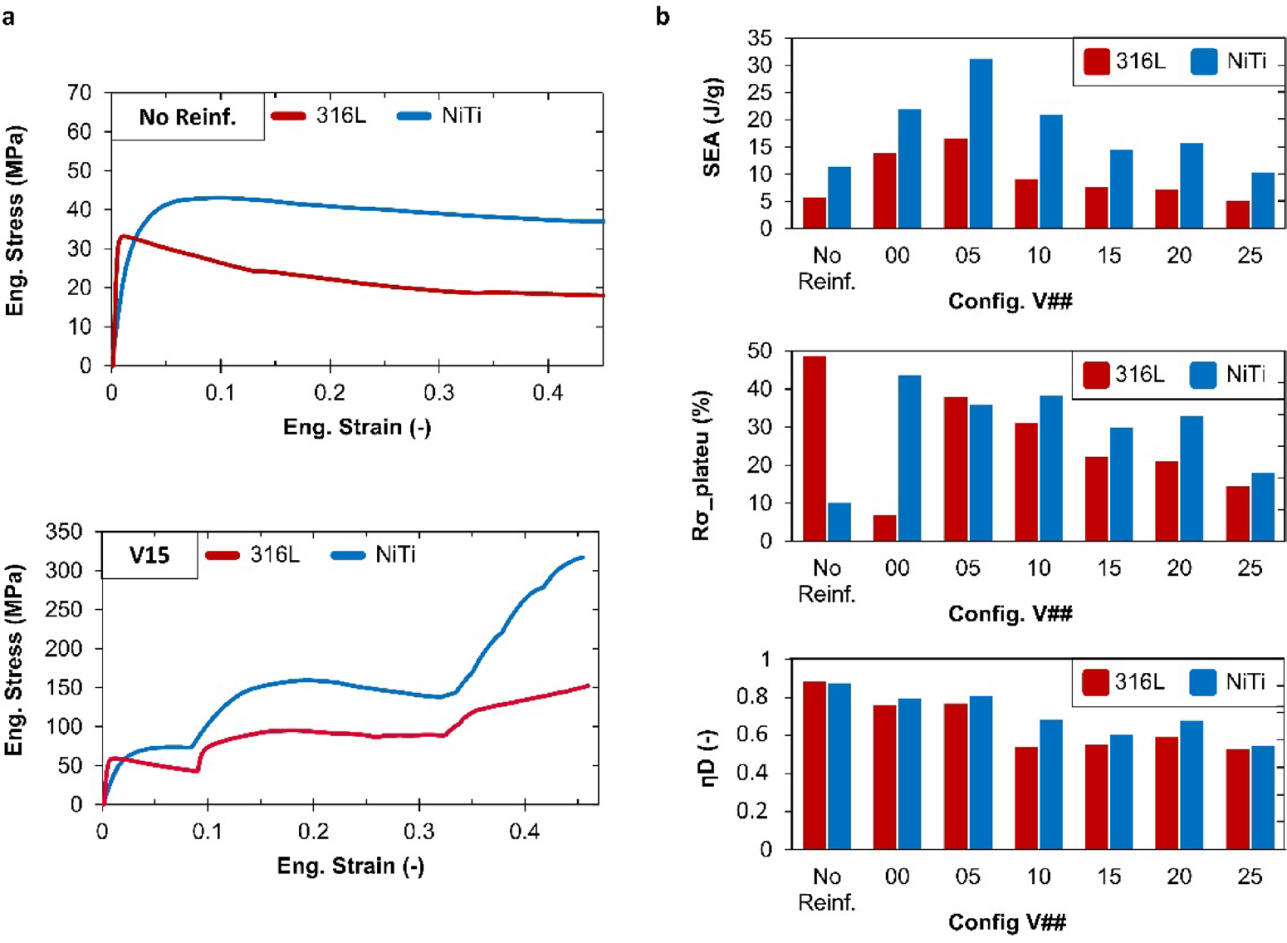
FEM results for stainless steel 316L and NiTi alloy unit cell: (**a**) demonstration of the same trend in eng. stress–strain curves progress; (**b**) main output metrics.

Figure 8a compares the eng. stress–strain curves from FEM analysis of NiTi alloy and stainless steel 316L, assuming the NiTi material model as a defect-free ductile material, similar to 316L. Configurations No Reinf. and V15 were chosen for demonstration purposes, as all other configurations shared the same trend. Samples from NiTi alloy showed an almost identical response to those from stainless steel, differing only in the higher absolute eng. stress value. As a result, NiTi alloy samples were able to absorb more energy (Fig. 8b), thus achieving higher *SEA* (approx. twice as high). The deviation from plateau characteristic (${R}_{\sigma \_plateau}$) and densification efficiency (${\eta}_{D}$) values were approx. 20% and 5% lower for stainless steel, respectively. Configurations No Reinf. and V00 were the only exceptions due to a different mode of lateral distortion captured in the simulations.

#### Reinforced unit cell assessment

The distance of the reinforcements from the cell centre was crucial for stable deformation and the amount of absorbed energy. Configurations with reinforcements at the centre of the cell should be avoided because of unstable deformation caused by lateral distortion. The risk of lateral distortion would be even higher as the number of cells increases. For the investigated configurations in this study, the range from $V$ ≈ 0.5 to 1.5 mm should be the most suitable, as it combines a high *SEA*, low deviation from the plateau characteristic, and stable deformation.

Obtained findings on the use of reinforcements point to their benefits not only in the field of energy absorbers. The secondary steep increase of the load capacity after contact of reinforcements and horizontal arms can be beneficial for ensuring the safety even after the first load capacity has been exceeded, e.g. in civil engineering and advanced concrete reinforcement^50^.

The results can be used as a guide to define the appropriate position of the reinforcement, but the exact values of the placement should be considered individually in the context of the structure type, the number of cells used, the ${t}_{A}/{t}_{R}$ ratio, loading conditions, etc.

From the obtained results, NiTi alloy would be a better choice for structures for energy absorption, as it achieved higher *SEA*, while having ${R}_{\sigma \_plateau}$ and ${\eta}_{D}$ similar to 316L. The same conclusion for cyclic loading is reported in^51^. However, the behaviour of additively manufactured NiTi alloy poses a serious obstacle to its reliable use in real-life applications, where high ductility is necessary. Therefore, a deeper insight into the mechanical properties of additively manufactured NiTi alloy is needed to better understand the influence of wall thickness on the material behaviour. Until that time, the full potential of NiTi alloy for energy absorption cannot be exploited, and stainless steel will be more suitable for a reliable, high-performance energy-absorbing structure.

### High-performance structure

#### Single parameter analysis

**Fig. 9:**
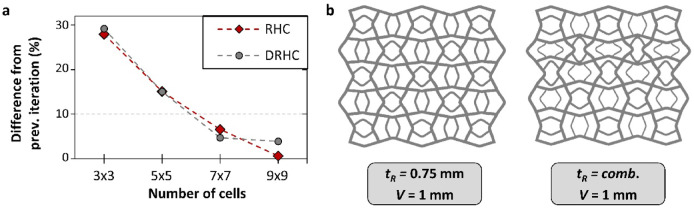
Overview of designing the high-performance structures: (**a**) number of cell independence; (**b**) influence of the reinforcement’s thickness *t*_*R*_.

Based on the criterion in section “High-performance auxetic structure” the 5 $\times$ 5 unit cell structure was chosen (Fig. 9a). Structures with ${R}_{A}={R}_{V}=$ 1.5 mm achieved higher *SEA* and lower deviation from the plateau characteristic compared to other configurations. A lower value of ${R}_{A}$ significantly increased the risk of unstable deformation pattern because the arms were oriented closer to the loading direction. On the other hand, higher values led to earlier contact between opposite arms, resulting in early densification and lower *SEA*. $V=$ 1 mm was chosen for the final geometry, as it was able to absorb more energy at a sufficiently low value of ${R}_{\sigma \_plateu}<$ 10% compared to other configurations. The effect of reinforcement placement was the same as discussed in section “Stainless steel 316L”. In terms of stability of the deformation, structures with ${t}_{R} =$ 0.75 mm and *combined* were the closest to the plateau characteristic (${R}_{\sigma \_plateu}<$ 10%). For uniform thickness ${t}_{R}=$ 0.75 mm, only horizontal reinforcements deformed (Fig. 9b*)*, whereas with ${t}_{R}=$ *combined* all reinforcements in one layer deformed simultaneously. Table 4 summarises the chosen parameters of the designed structures and a reference structure without reinforcements. These configurations were manufactured from stainless steel 316L and then tested.

**Table 4:** Designed high-performance structures with 5 × 5 cells (*R*_*A*_ = 1.5 mm; *t*_*A*_ = 1.0 mm).

Configuration	S0	S1	S2
*V* (mm)	–	1.0	1.0
*t*_*R*_ (mm)	–	0.75	0.5 + 0.75
Model			

#### FEM analysis and experimental validation

**Fig. 10:**
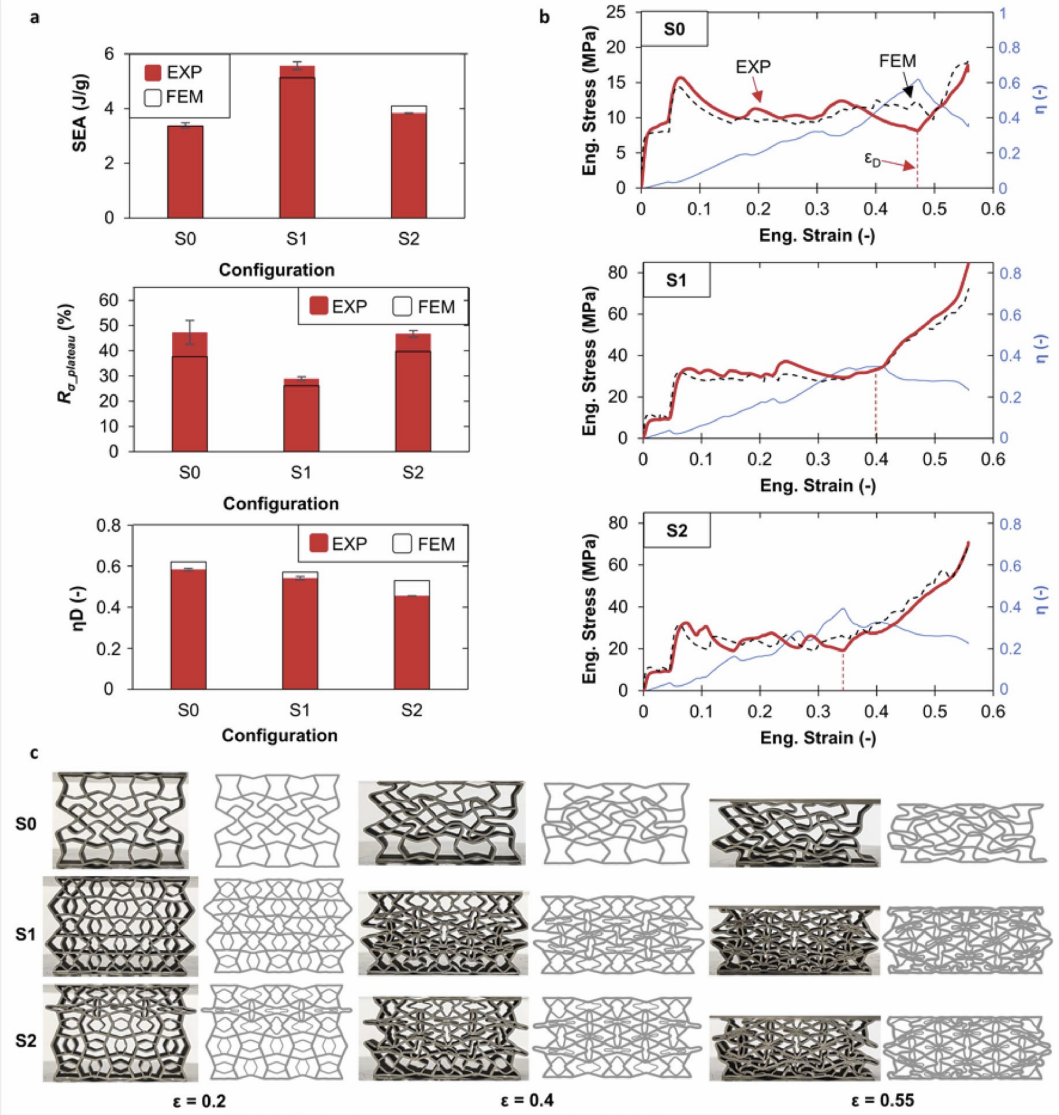
(**a**) Summary of the key output metrics for experimental and FEM testing; (**b**) Eng. stress–strain curves (experimental + FEM) and energy absorption efficiency (experimental); (**c**) Deformation process comparison for experiment and FEM simulations.

The obtained results confirmed the benefits of reinforcements in the DRHC structure, as shown in Fig. 10a. The results of quasi-static compression tests showed a very good agreement between experiment and FEM simulations, in both the deformation and eng. stress–strain curve progress.

The S1 configuration achieved homogenous stiffness in all sections, leading to a smooth deformation and the best performance (Fig. 10b). Its *SEA* reached 5.47 J/g at a densification strain of 0.40, with a plateau stress of 32.06 MPa. Compared to the S0 reference geometry, the *SEA* was more than 70% higher, while achieving a significantly smaller deviation from the plateau characteristic (17.67% for S1, 47.07% for S0). Therefore, the uniform deformation of S1 compensated its earlier densification. However, the maximal energy absorption efficiency ${\eta}_{\mathrm{m}\mathrm{a}\mathrm{x}}$ of the S1 structures was significantly lower: 0.347 for S1, 0.616 for S0.

The eng. stress–strain curves of the tested configurations are shown in Fig. 10b. A detailed analysis of the σ(ε) curve of the best-performing structure S1 is shown in the Supplementary Material. The fluctuation in the σ(ε) of S1 was caused by the layer-by-layer deformation. The compression process was similar for S2, but the deformation of the actual layer did not end at the moment the reinforcements touched the arms. However, due to the designed different thicknesses of the horizontal and vertical reinforcements, the row was fully compressed (Fig. 10c). This resulted in greater fluctuation in eng. stress and thus reduced absorption performance. Therefore, a similar thickness of reinforcements and arms (${t}_{R} \approx {t}_{A}$) is better for effective energy dissipation. For S0, these fluctuations were partially damped by lateral distortion of the structure.

It was found that only the middle three layers were fully compressed at the densification for all configurations. Therefore, the value of densification was relatively low (${\varepsilon}_{D}\approx 0.4; {\eta}_{D}\approx 0.5$). The outer two layers, which were in direct contact with the compression plates, were not allowed to deform in the same way as the inner rows. Thus, it can be expected that as the number of cells in the loading direction increases, the influence of the upper and bottom rows would become less significant and densification could be achieved at eng. strain values greater than 50%.

**Fig. 11:**
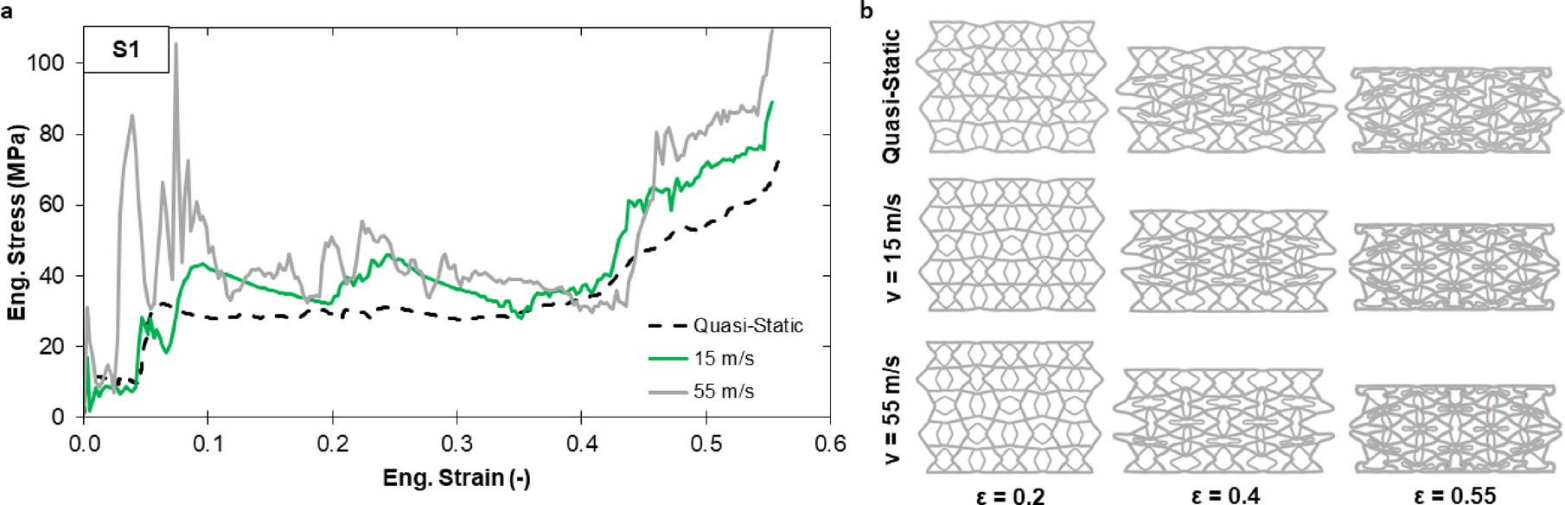
(**a**) Eng. stress–strain curves (FEM) for quasi-static and dynamic loading (velocities of 15 m/s and 55 m/s); (**b**) Deformation pattern during compression up to the fully densified state.

Dynamic compression tests for the S1 configuration confirmed a stable deformation process for both impact velocities (15 m/s and 55 m/s) with an identical densification pattern as for quasi-static compression (Fig. 11). Inertia and strain-hardening effects of the material became significant under dynamic loading conditions, resulting in higher peak and plateau eng. stress, later densification, higher *SEA* and layer-by-layer deformation process starting at the top (impact) layer. All of these correspond with recent literature^42^. The *SEA* for quasi-static compression and at impact velocities of 15 m/s and 55 m/s were 5.13 J/g, 6.85 J/g and 8.62 J/g, respectively, while the peak eng. stress was 34.6 MPa, 45.92 MPa and 105.52 MPa, respectively. Extreme fluctuations of eng. stress at impact velocity of 55 m/s resulted in 155.37% value of ${R}_{\sigma \_plateau}$ (26.13% for quasi-static). The densification values for impact load were slightly higher: 0.428 for 15 m/s and 0.435 for 55 m/s, compared to 0.413 for quasi-static compression. Moreover, the energy absorption efficiency was significantly higher under dynamic loading: 15 m/s and 55 m/s achieved 0.413 and 0.573, respectively, whereas quasi-static loading achieved only 0.327.

#### High-performance structures assessment

**Fig. 12:**
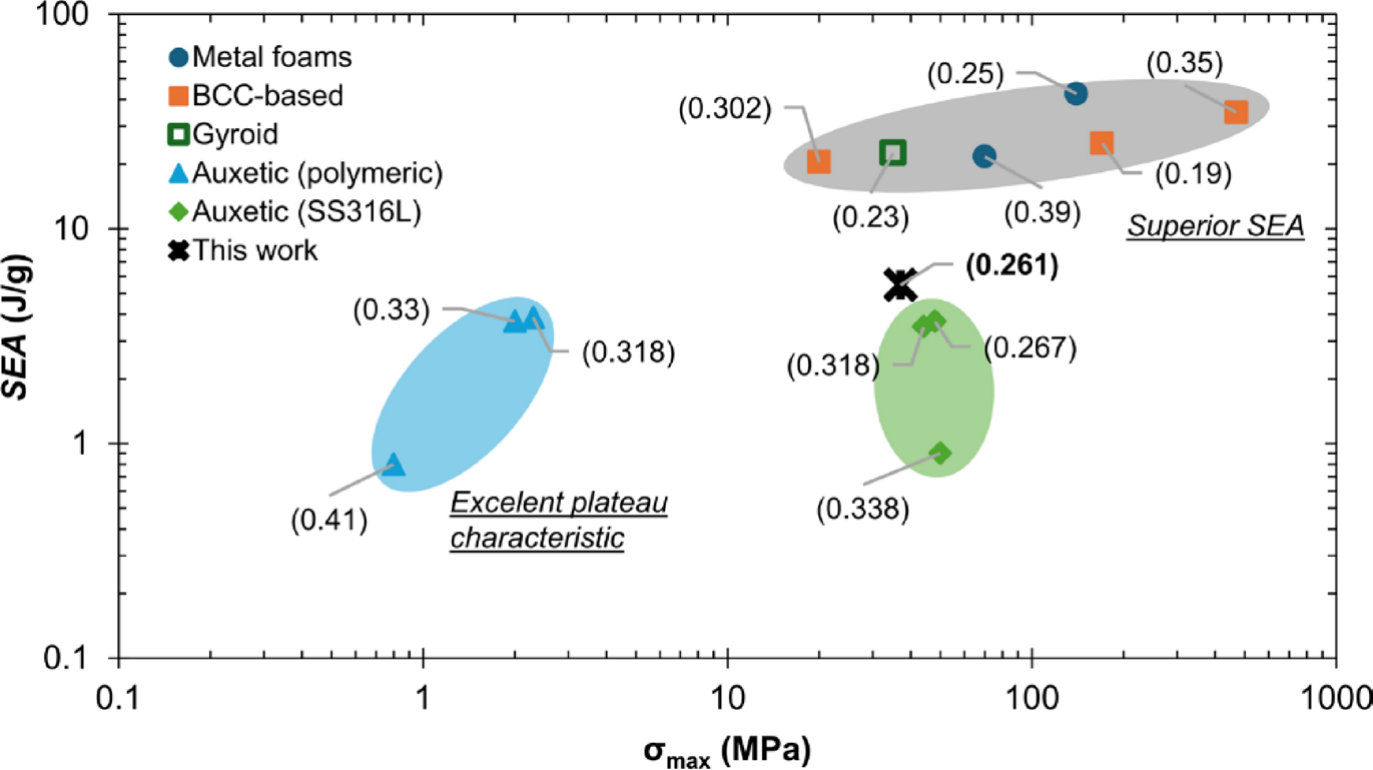
Comparison of S1 configuration with other relevant metamaterials by *SEA* and peak eng. stress, values in parentheses show relative density (data are from^2,18,19,28–33,52–54^).

When compared with structures mentioned in section “High-performance structures” and auxetic structures from stainless steel^52–54^ (Fig. 12), the S1 configuration exhibits enhanced absorption performance in both *SEA* and stability of deformation. Implementation of reinforcements was beneficial for structures, but they were very sensitive to the parameters ${R}_{A}, V \mathrm{a}\mathrm{n}\mathrm{d} {t}_{R}$, resulting in a high chance of lateral distortion. Approximately the same stiffness of all regions of configuration S1 caused evenly distributed load across the whole structure, resulting in the best performance. However, it should be possible to reach even better results if the densification occurs later. This could be achieved by increasing the number of cells in the direction of the applied force, so that the outermost row of the structure has a negligible impact on the densification value.

There is still some flexibility to further adjust the mechanical properties of the structure by changing the thickness of the arms ${t}_{A}$. However, the new values of ${t}_{A}$ should be close to the tested value of 1 mm, while ${t}_{R}$ should remain at scale. This is because a smaller ${t}_{A}$ could change the deformation mechanism to a twisting collapse of the arms instead of their rotation around plastic hinges. For a larger thickness, the arms could behave as a bulk material and no longer deflect inward, so the reinforcement would not be used efficiently.

## Conclusions

This study investigated and systematically designed an additively manufactured auxetic structure using FEM analysis and verified it through a quasi-static compression test. The main achievement is the explanation of the consequences of implementing the reinforcements on the formation of the plastic hinge and on energy absorption performance. Using the obtained findings, a high-performance auxetic structure was designed that achieved enhanced properties compared to a reference structure. The main findings of this study are as follows:

- Reinforcing the RHC cell resulted in up to three times higher load capacity and *SEA* value, when the most suitable position of the reinforcements was near the centre of the cell (V ≈ 0.5 to 1.5 mm).
- The secondary steep increase in load capacity that occurs when the reinforcements contact the horizontal arms can provide additional safety in civil engineering applications, once the initial load capacity has been exceeded.
- Incorrect placement of reinforcements significantly disrupted the homogeneity of the material distribution, causing unstable deformation due to regions of different stiffness.
- The assumption of plastic hinge formation at the connection points of the reinforcement to the arms was disproved, based on the obtained distribution of the plasticised material.
- The Double Re-entrant Honeycomb structure showed an optimal deformation pattern for the use of reinforcements and stable deformation close to the plateau characteristic.
- The newly designed high-performance structure absorbed more than 70% energy per unit mass, while decreasing the engineering stress fluctuations by about 36% compared to the reference structure without reinforcements.
- The deformation pattern of the newly designed structure remained the same for both quasi-static and dynamic loading conditions, demonstrating a reliable and stable response.
- NiTi alloy would perform better than stainless steel 316L, but its brittle behaviour due to insufficient process parameters is a serious obstacle.

The presented findings can be used to design new structures and modify existing ones in a more targeted manner to achieve efficient energy dissipation and high absorption performance. Further research of the observed issue could investigate the combination of methods mentioned in the literature, leading to a further increase in absorbed energy, or the behaviour of the structure with reinforcements under general loading conditions.

## Supplementary Information

Below is the link to the electronic supplementary material.

Supplementary Material 1

Supplementary Material 2

Supplementary Material 3

Supplementary Material 4

## Data Availability

The data that support the findings of this study are openly available in Zenodo repository at 10.5281/zenodo.17157279. The preprint for this study is openly available in Zenodo repository at 10.5281/zenodo.17206332.

## Abbreviations

2D: Two-dimensional
BCC: Body centred cubic
CAD: Computer aided design
CoF: Coeficient of friction
DIC: Digital image correlation
DRHC: Double re-entrant honeycomb
EXP: Experiment
FEM: Finite element method
LPBF: Laser powder bed fusion
RHC: Re-entrant honeycomb
TPMS: Triply periodic minimal surface
${E}_{\mathrm{S}}$: Elastic modulus of bulk material
$${E}_{\mathrm{t}}$$: Tangent modulus
$$F$$: Reaction force
${R}_{\mathrm{m}}$: Ultimate tensile strength
${R}_{\mathrm{p}\mathrm{0,2}}$: Yield strength (0.2% proof stress)
${R}_{A}$: Deflection of arm
${R}_{R}$: Deflection of reinforcement
${R}_{{\sigma{\_plateau}}}$: Deviation from plateau characteristic
${S}_{str}$: Area of specimen projection in loading direction
*SEA*: Specific energy absorption
$${t}_{A}$$: Arm thickness
$${t}_{R}$$: Reinforcement thickness
$V$: Position of reinforcement
*x*_*0*_, *y*_*0*_: Initial dimensions in the direction of the coordinate axis
$y$: Displacement in *y*-direction
$$\varepsilon$$: Engineering strain
$${\varepsilon}_{D}$$: Densification
$\eta , {\eta}_{\mathrm{m}\mathrm{a}\mathrm{x}}$: Energy absorption efficiency, maximal energy absorption efficiency
${\eta}_{D}$: Efficiency of densification
$$\upmu$$: Poisson’s number
${\rho}_{\mathrm{R}\mathrm{E}\mathrm{L}}$: Measured relative density
${\rho}_{\mathrm{S}}$: Density of bulk material
$\upsigma , {\upsigma}_{\mathrm{m}\mathrm{a}\mathrm{x}}$: Engineering stress, maximal engineering stress
$${\upsigma}_{plateau}$$: Plateau stress

## References

[1] Shinde, M. et al. Towards an ideal energy absorber: Relating failure mechanisms and energy absorption metrics in additively manufactured AlSi10Mg cellular structures under quasistatic compression. *J. Manuf. Mater. Process.*10.3390/jmmp6060140 (2022).

[2] Yuan, S., Chua, C. K. & Zhou, K. 3D-Printed mechanical metamaterials with high energy absorption. *Adv. Mater. Technol.*10.1002/admt.201800419 (2019).

[3] Lu, G. & Yu, T. X. *Energy Absorption of Structures and Materials* (Woodhead Pub., 2003).

[4] Gibson, L. J. & Ashby, M. F. *Cellular Solids: Structure and Properties* (Cambridge University Press, 1997).

[5] Ren, X., Das, R., Tran, P., Ngo, T. D. & Xie, Y. M. Auxetic metamaterials and structures: A review. *Smart Mater. Struct.*. 10.1088/1361-665X/aaa61c (2018).

[6] Zhou, X. et al. Advances in 3D/4D printing of mechanical metamaterials: From manufacturing to applications. *Compos. Part B Eng.* **254**, 110585. 10.1016/j.compositesb.2023.110585 (2023).

[7] Zhang, L. et al. Decoupling microlattice metamaterial properties through a structural design strategy inspired by the Hall–Petch relation. *Acta Mater.*10.1016/j.actamat.2022.118214 (2022).37599815

[8] Saxena, K. K., Das, R. & Calius, E. P. Three decades of auxetics research—materials with negative Poisson’s ratio: A review. *Adv. Eng. Mater.* **18**, 1847–1870. 10.1002/adem.201600053 (2016).

[9] Evans, K. E. & Alderson, A. Auxetic materials: Functional materials and structures from lateral thinking!. *Adv. Mater.* **12**, 617–628 (2000).

[10] Scarpa, F., Giacomin, J. A., Bezazi, A. & Bullough, W. A. Dynamic behavior and damping capacity of auxetic foam pads. In *Smart Structures and Materials 2006: Damping and Isolation* vol. 6169 61690T (SPIE, 2006).

[11] Zhang, J., Lu, G. & You, Z. Large deformation and energy absorption of additively manufactured auxetic materials and structures: A review. *Compos. Part B Eng..*10.1016/j.compositesb.2020.108340 (2020).

[12] Evans, K. E. The design of doubly curved sandwich panels with honeycomb cores. *Compos. Struct.*10.1016/0263-8223(91)90064-6 (1991).

[13] Duncan, O. et al. Review of auxetic materials for sports applications: Expanding options in comfort and protection. *Appl. Sci.*10.3390/app8060941 (2018).

[14] Alderson, A., Alderson, K. L., Chirima, G., Ravirala, N. & Zied, K. M. The in-plane linear elastic constants and out-of-plane bending of 3-coordinated ligament and cylinder-ligament honeycombs. *Compos. Sci. Technol.* **70**, 1034–1041 (2010).

[15] Li, T., Liu, F. & Wang, L. Enhancing indentation and impact resistance in auxetic composite materials. *Compos. Part B Eng.*10.1016/j.compositesb.2020.108229 (2020).

[16] Liu, W., Wang, N., Luo, T. & Lin, Z. In-plane dynamic crushing of re-entrant auxetic cellular structure. *Mater. Des.* **100**, 84–91 (2016).

[17] Li, Z., Wang, K. F. & Wang, B. L. Indentation resistance of brittle auxetic structures: Combining discrete representation and continuum model. *Eng. Fract. Mech.*10.1016/j.engfracmech.2021.107824 (2021).

[18] Xie, B., Fan, Y. Z., Mu, T. Z. & Deng, B. Fabrication and energy absorption properties of titanium foam with CaCl_2_ as a space holder. *Mater. Sci. Eng. A* **708**, 419–423 (2017).

[19] Choy, S. Y., Sun, C. N., Leong, K. F. & Wei, J. Compressive properties of functionally graded lattice structures manufactured by selective laser melting. *Mater. Des.* **131**, 112–120 (2017).

[20] Jia, Z., Liu, F., Jiang, X. & Wang, L. Engineering lattice metamaterials for extreme property, programmability, and multifunctionality. *J. Appl. Phys.*10.1063/5.0004724 (2020).

[21] Červinek, O. et al. Computational approaches of quasi-static compression loading of SS316L lattice structures made by selective laser melting. *Materials*10.3390/ma14092462 (2021). 34068584 10.3390/ma14092462 PMC8126075

[22] Yi, J. et al. Unleashing multi-scale mechanical enhancement in NiTi shape memory alloy via annular intra-laser deposition with homogenized Ti2Ni nanoprecipitates. *Acta Mater.*10.1016/j.actamat.2023.119418 (2024).

[23] Cherouat, A., Barriere, T. & Wang, H. Experimental analysis of extrusion-based additive manufacturing process of bio-composite NiTi alloy. *Int. J. Damage Mech.* **34**, 573–597 (2025).

[24] Mohd Jani, J., Leary, M., Subic, A. & Gibson, M. A. A review of shape memory alloy research, applications and opportunities. *Mater. Design* **56**, 1078–1113. 10.1016/j.matdes.2013.11.084 (2014).

[25] Sun, L. et al. Mechanical and shape memory properties of NiTi triply periodic minimal surface structures fabricated by laser powder bed fusion. *J. Manuf. Process.* **101**, 1091–1100 (2023).

[26] Zhou, M. et al. NiTi alloy helical lattice structure with high reusable energy absorption and enhanced damage tolerance. *J. Mater. Sci. Technol.* **217**, 237–244 (2025).

[27] Li, Q. M., Magkiriadis, I. & Harrigan, J. J. Compressive strain at the onset of densification of cellular solids. *J. Cell. Plast.* **42**, 371–392 (2006).

[28] Sharma, D. & Hiremath, S. S. Bio-inspired repeatable lattice structures for energy absorption: Experimental and finite element study. *Compos. Struct.*10.1016/j.compstruct.2021.115102 (2022).

[29] Stanev, L. M. et al. Compressive properties and energy absorption behaviour of AlSi10Mg open-cell foam surface modification of aluminum and aluminum alloys with nanoparticles by means of intensity energy flows view project STUDY OF NANO-MODIFIED COATINGS PRODUCED BY MANUAL ARC OVERLAY WELDING view project compressive properties and energy absorption behaviour of AlSi10Mg open-cell foam. *J. Mater. Sci. Technol.* **22**, 44–53 (2014).

[30] Yang, Z., Liu, H., Wu, Q. & Long, L. Study of energy absorption characteristics and deformation mechanism of stretching–bending synergistic lattices under dynamic compression. *Adv. Eng. Mater.*10.1002/adem.202201130 (2023).

[31] Maskery, I., Aboulkhair, N. T., Aremu, A. O., Tuck, C. J. & Ashcroft, I. A. Compressive failure modes and energy absorption in additively manufactured double gyroid lattices. *Addit. Manuf.* **16**, 24–29 (2017).

[32] Najafi, M., Ahmadi, H. & Liaghat, G. Experimental investigation on energy absorption of auxetic structures. In *Materials Today: Proceedings* vol. 34 350–355 (Elsevier Ltd, 2019).

[33] Dong, J., Ye, G., Wang, Y., Jin, F. & Fan, H. Design, manufacture and crushing behaviors of buckling-inspired auxetic meta-lattice structures. *Int. J. Smart Nano Mater.* **12**, 491–510 (2021).

[34] Gao, Q. & Liao, W. H. Energy absorption of thin walled tube filled with gradient auxetic structures-theory and simulation. *Int. J. Mech. Sci.*10.1016/j.ijmecsci.2021.106475 (2021).

[35] Lim, T. C. A 3D auxetic material based on intersecting double arrowheads. *Phys. Status Solidi B Basic Res.* **253**, 1252–1260 (2016).

[36] Gohar, S., Hussain, G., Ilyas, M. & Ali, A. Performance of 3D printed topologically optimized novel auxetic structures under compressive loading: experimental and FE analyses. *J. Mater. Res. Technol.* **15**, 394–408 (2021).

[37] Tancogne-Dejean, T., Spierings, A. B. & Mohr, D. Additively-manufactured metallic micro-lattice materials for high specific energy absorption under static and dynamic loading. *Acta Mater.* **116**, 14–28 (2016).

[38] Wang, P., Yang, F., Li, P., Zheng, B. & Fan, H. Design and additive manufacturing of a modified face-centered cubic lattice with enhanced energy absorption capability. *Extreme Mech. Lett.*10.1016/j.eml.2021.101358 (2021).

[39] Li, D., Yin, J., Dong, L. & Lakes, R. S. Strong re-entrant cellular structures with negative Poisson’s ratio. *J. Mater. Sci.* **53**, 3493–3499 (2018).

[40] Li, F., Zhang, Q., Shi, H. & Liu, Z. A modified three-dimensional negative-Poisson-ratio metal metamaterial lattice structure. *Materials*10.3390/ma15113752 (2022). 35683059 10.3390/ma15113752 PMC9181437

[41] Zhang, X. et al. Quasi-static compression and dynamic crushing behaviors of novel hybrid re-entrant auxetic metamaterials with enhanced energy-absorption. *Compos. Struct.*10.1016/j.compstruct.2022.115399 (2022).

[42] Wang, E. et al. Numerical and constitutive modeling of quasi-static and dynamic mechanical behavior in graded additively manufactured lattice structures. *Virtual Phys. Prototyp.*10.1080/17452759.2023.2283027 (2023).

[43] Jaros, J., Sobol, V. & Červinek, O. Mechanical properties of PBF-LB NiTi under quasi-static and cyclic compressive loading at low speed.

[44] Červinek O., J. J. , K. D. Process parameters for NiTi alloy manufactured by Laser Powder Bed Fusion. 10.5281/zenodo.14499514 (2025).

[45] Cervinek, O., Pettermann, H., Todt, M., Koutny, D. & Vaverka, O. Non-linear dynamic finite element analysis of micro-strut lattice structures made by laser powder bed fusion. *J. Mater. Res. Technol.* **18**, 3684–3699 (2022).

[46] Vivegananthan, P. et al. Recent progress on 3D printing of lightweight metal thin-walled structures. *Adv. Mater.* **36**, 2402130 (2024). 10.1002/adma.202402130 39420709

[47] Wrobel, R. et al. Influence of wall thickness on microstructure and mechanical properties of thin-walled 316L stainless steel produced by laser powder bed fusion. *Mater. Des.* **238**, 112652 (2024).

[48] Brown, B., Everhart, W. & Dinardo, J. Characterization of bulk to thin wall mechanical response transition in powder bed AM. *Rapid Prototyp. J.* **22**, 801–809 (2016).

[49] Li, K. et al. Insight into the cracking mechanism of super-elastic NiTi alloy fabricated by laser powder bed fusion. *Virtual Phys. Prototyp.*10.1080/17452759.2024.2368654 (2024).

[50] Vandadi, M., Heidarnezhad, S., Pourhaji, P. & Rahbar, N. Integrating 3D-printed auxetic structures for advanced concrete reinforcement. *Adv. Mater. Interfaces* **12**, e00095 (2025).

[51] Moutablaleh, H., Abdelhady, E. S., Vaneker, T., Gibson, I. & Mehrpouya, M. A comparative analysis of functional performance in additively manufactured NiTi, Ti-6Al-4V, and 316L stainless steel architected metastructures. *J. Mech. Behav. Biomed. Mater.* **169**, 107071 (2025). 40412349 10.1016/j.jmbbm.2025.107071

[52] Zhang, Y. et al. In-plane compressive properties of assembled auxetic chiral honeycomb composed of slotted wave plate. *Mater. Des.* **221**, 110956 (2022).

[53] Zhang, W., Wang, H., Li, X. & Shao, J. In-plane crushing behavior and energy absorption of novel X-shaped auxetic metamaterial: Experimental and numerical investigations. *J. Mater. Sci.* **60**, 11048–11074 (2025).

[54] Wang, Z., Zhang, J., Kong, S. & Zhu, Y. 3D-printed re-entrant auxetic metamaterials with bi-directional stiffness enhancement. *Thin-Walled Struct.* **216**, 113722 (2025).

