# Supplementary material for: Comprehensive study of the influence of reinforcements on the deformation mechanism and energy absorption performance of auxetic structure
Source: Sci Rep. 2026 May 19;16:22844. doi: 10.1038/s41598-026-53790-z (PMC13389475; doi:10.1038/s41598-026-53790-z)
Supplement: Supplementary file 1 — Supplementary Material 1 [file 41598_2026_53790_MOESM1_ESM.pdf]

SUPPLEMENTARY MATERIAL

to article

Comprehensive Study of the Influence of Reinforcements on the Deformation Mechanism and Energy Absorption Performance of Auxetic Structure

Vítězslav Sobol [1]*, Ondřej Červinek [1], Jan Jaroš [1], Melanie Todt [2], Jakub Hurník [1], Daniel Koutný [1]

[1] Brno University of Technology, Faculty of Mechanical Engineering, Institute of Machine and Industrial Design, Technická 2896/2, 616 69 Brno, Czech Republic

[2] Technische Universitaet Wien, Institute of Lightweight Design and Structural Biomechanics, Karlsplatz 13, 1040 Vienna, Austria

*Corresponding author's email: Vitezslav.Sobol@vut.cz (V. Sobol)

Supplement A

The obtained force-displacement curves from all numerical and experimental analyses were transformed into eng. stress-strain curve according to $\sigma = F/S_{str}$, where σ is eng. stress, F is obtained reaction force, S_{str} is the area of specimen projection in the direction of the force; and $\varepsilon = y/y_0$, where ε is eng. strain, y is the actual compression value, and y_0 is the initial height of the specimen. Output parameters derived from eng. stress-strain curves were relative density (ρ_{REL}), energy absorption efficiency (η), onset of densification (ε_D), densification efficiency (η_D), Specific Energy Absorption (*SEA*), and deviation from plateau characteristic ($R_{\sigma\_plateau}$). Their descriptions are listed below.

Onset of densification ($\boldsymbol{\varepsilon_D}$) was set at the point of the highest **energy absorption efficiency ($\boldsymbol{\eta}$)** value according to (A.1) and (A.2) [1], where σ and ε are eng. stress and eng. strain, respectively.

$$\varepsilon_D = \varepsilon(\eta_{\max}) \tag{A.1}$$

$$\eta(\varepsilon) = \frac{1}{\sigma(\varepsilon)} \int_0^{\varepsilon} \sigma(\varepsilon)\,\mathrm{d}\varepsilon \quad (-) \tag{A.2}$$

Densification efficiency ($\boldsymbol{\eta_D}$) was used to assess the reached moment of densification. It was calculated using formula (A.3) [2]. **Relative density ($\boldsymbol{\rho_{\mathrm{REL}}}$)** is given by (A.4), where m^* is measured mass, ρ_{S} is material density and V_{str} is volume of envelope cuboid.

$$\eta_D = \frac{\varepsilon_D}{1 - \rho_{\mathrm{REL}}} \quad (-). \tag{A.3}$$

$$\rho_{\mathrm{REL}} = \frac{m^*}{\rho_{\mathrm{S}} V_{\mathrm{str}}} \quad (-) \tag{A.4}$$

Specific energy absorption (*SEA*) was determined according to formula (A.5) [3] from the $\sigma(\varepsilon)$ curve and measured relative density.

$$SEA = \frac{\int_0^{\varepsilon_D} \sigma(\varepsilon)\,\mathrm{d}\varepsilon}{\rho_{\mathrm{S}}\,\rho_{\mathrm{REL}}} \quad (\mathrm{J\,kg^{-1}}). \tag{A.5}$$

Plateau stress ($\boldsymbol{\sigma_{plateau}}$) was calculated as the average value of the eng. stress in the region between the first eng. stress peak and the onset of densification ε_D.

Deviation from plateau characteristic ($R_{\sigma\_plateau}$) was used to quantify the amount of potentially absorbed energy with ideal plateau characteristic compared to really absorbed one. The value was evaluated using (A.6), where $\sigma_{\max}$ is maximum eng. stress before densification ε_D:

$$R_{\sigma\_plateau} = \left(\frac{\sigma_{\max}\,\varepsilon_D}{\int_0^{\varepsilon_D} \sigma(\varepsilon)\,\mathrm{d}\varepsilon} - 1 \right) \cdot 100 \quad (\%). \tag{A.6}$$

Data from the **DIC camera recordings** were used for single cell with reinforcements to create strain maps, in the form of *Eng. Equivalent Strain (von Mises)*. Furthermore, recordings were used to see the deformation process to pick up critical moments without any measurable variable.

Supplement B

Table B.1. Output parameters from experimental test for unit cells from stainless steel 316L.

Configuration		ρ_{REL} (-)	*SEA* (J/g)	$R_{\sigma\_plateau}$ (%)	η_D (-)	σ_{max} (MPa)	ε_D (-)
	V00	0.372	9.22	28.27	0.63	89.27	0.392
	V05	0.382	16.93	41.72	0.71	165.95	0.439
**316L**	V10	0.386	14.84	58.74	0.72	163.48	0.442
**-**	V15	0.383	8.00	32.69	0.53	98.70	0.327
**EXP**	V20	0.392	6.51	27.89	0.54	79.58	0.326
	V25	0.398	5.31	29.66	0.52	70.14	0.311
	No Reinf.	0.279	5.45	55.29	0.76	34.14	0.550

Table B.2. Output parameters from experimental test for unit cells from NiTi alloy (all parameters are evaluated up to break of the sample, approx. 10 % of eng. strain).

Configuration		ρ_{REL} (-)	*SEA* (J/g)	$R_{\sigma\_plateau}$ (%)	η_D (-)	σ_{max} (MPa)	ε_D (-)
	V00	0.431	2.79	72.45	0.16	143.00	0.092
	V05	0.440	1.83	53.23	0.13	104.41	0.075
**NiTi**	V10	0.444	1.03	56.50	0.10	82.74	0.055
**-**	V15	0.447	1.74	38.87	0.17	73.12	0.094
**EXP**	V20	0.451	2.01	44.87	0.19	81.15	0.103
	V25	0.454	1.85	47.59	0.16	87.34	0.090
	No Reinf.	0.325	0.46	53.33	0.06	38.32	0.037

Table B.3. Output parameters from FEM analysis for unit cells from stainless steel 316L.

Configuration		ρ_{REL} (-)	*SEA* (J/g)	$R_{\sigma\_plateau}$ (%)	η_D (-)	σ_{max} (MPa)	ε_D (-)
	V00	0.399	13.83	6.83	0.76	102.60	0.457
	V05	0.406	16.63	37.84	0.77	162.03	0.457
**316L**	V10	0.410	13.96	43.60	0.74	149.09	0.438
**-**	V15	0.413	7.66	22.05	0.55	94.99	0.323
**FEM**	V20	0.417	7.21	21.06	0.59	84.18	0.344
	V25	0.421	5.11	14.37	0.53	63.92	0.306
	No Reinf.	0.306	5.09	34.40	0.85	28.25	0.589

Table B.4. Output parameters from FEM analysis for unit cells from NiTi alloy (all parameters are evaluated up to full compression).

Configuration		ρ_{REL} (-)	*SEA* (J/g)	$R_{\sigma\_plateau}$ (%)	η_D (-)	σ_{max} (MPa)	ε_D (-)
	V00	0.428	21.92	43.60	0.79	187.84	0.455
	V05	0.438	31.26	35.81	0.81	259.18	0.455
**NiTi**	V10	0.441	20.95	38.27	0.68	212.87	0.381
**-**	V15	0.446	14.50	29.81	0.60	159.23	0.334
**FEM**	V20	0.449	15.73	32.95	0.68	159.00	0.374
	V25	0.452	10.36	18.08	0.55	116.91	0.300
	No Reinf.	0.323	11.44	10.09	0.87	43.56	0.592

Supplement C

Table C.1. Output parameters from quasi-static experimental test for structures from stainless steel 316L.

Configuration		ρ_{REL} (-)	*SEA* (J/g)	$R_{\sigma\_plateau}$ (%)	η_D (-)	$\sigma_{plateau}$ (MPa)	σ_{max} (MPa)	ε_D (-)	η_{max} (-)
**316L**	S0	0.188	3.37	47.07	0.58	10.90	15.69	0.471	0.616
**-**	S1	0.261	5.47	17.67	0.49	32.06	37.20	0.401	0.347
**EXP**	S2	0.248	3.84	46.734	0.46	24.45	32.41	0.343	0.392

Table C.2. Output parameters from quasistatic FEM analysis for structures from stainless steel 316L.

Configuration		ρ_{REL} (-)	*SEA* (J/g)	$R_{\sigma\_plateau}$ (%)	η_D (-)	$\sigma_{plateau}$ (MPa)	σ_{max} (MPa)	ε_D (-)	η_{max} (-)
**316L**	S0	0.195	3.36	37.68	0.62	10.63	14.38	0.498	0.533
**-**	S1	0.277	5.13	26.13	0.57	29.75	34.60	0.413	0.327
**FEM**	S2	0.266	4.10	39.73	0.53	23.95	31.36	0.386	0.331

Table C.3. Output parameters from FEM analysis for structure S1 from stainless steel 316L for quasi-static and impact load.

Configuration		ρ_{REL} (-)	SEA (J/g)	$R_{\sigma\_plateau}$ (%)	η_D (-)	$\sigma_{plateau}$ (MPa)	σ_{max} (MPa)	ε_D (-)	η_{max} (-)
**316L**	Quasi-static	0.277	5.13	26.13	0.57	29.75	34.60	0.413	0.327
**-**	15 m/s	0.277	6.85	37.53	0.58	37.09	45.92	0.428	0.413
**FEM**	55 m/s	0.277	8.62	155.37	0.59	41.50	105.52	0.435	0.573

Supplement D

A detailed analysis of the σ(ε) curve of the best-performing structure S1 is shown in **Figure D1**. The description of the deformation process in **Figure D1** is given below:

0. Original state before loading.
1. Alignment of the upper and bottom outer arms leading to settlement of the upper plate on the main body of the structure.
2. Overcoming the load capacity of the structure followed by layer-by-layer deformation with a slight decrease in the eng. stress. The first layer involved was the very bottom row, in which the horizontal reinforcements deformed, allowing the arms to deflect.
3. Deformation of the horizontal reinforcements in the bottom row to its extreme position (contact with the arms), leading to an increase in the stiffness of these cells. This led to an increase in σ and deformation of a new row.
4. Repetition of steps 2 and 3 for other layers.
5. In all rows, the horizontal reinforcements had already deformed to their extreme position. The structure's load capacity was now dependent on the stiffness of the vertical reinforcements.
6. After a slight increase in the eng. stress, the vertical reinforcements also started to deform. This time, it was simultaneous for the three middle rows, as reflected in the smooth progression of the eng. stress up to the point of densification.
7. Full compression of the middle rows. Steep increase in σ and onset of the densification ε_D.
8. Full compression of all layers. An even steeper increase in the eng. stress resulting from the contact of all arms and reinforcements. Further displacement of the compression plate thus led to a compression of the base material rather than the structure geometry itself.
9. Test termination.

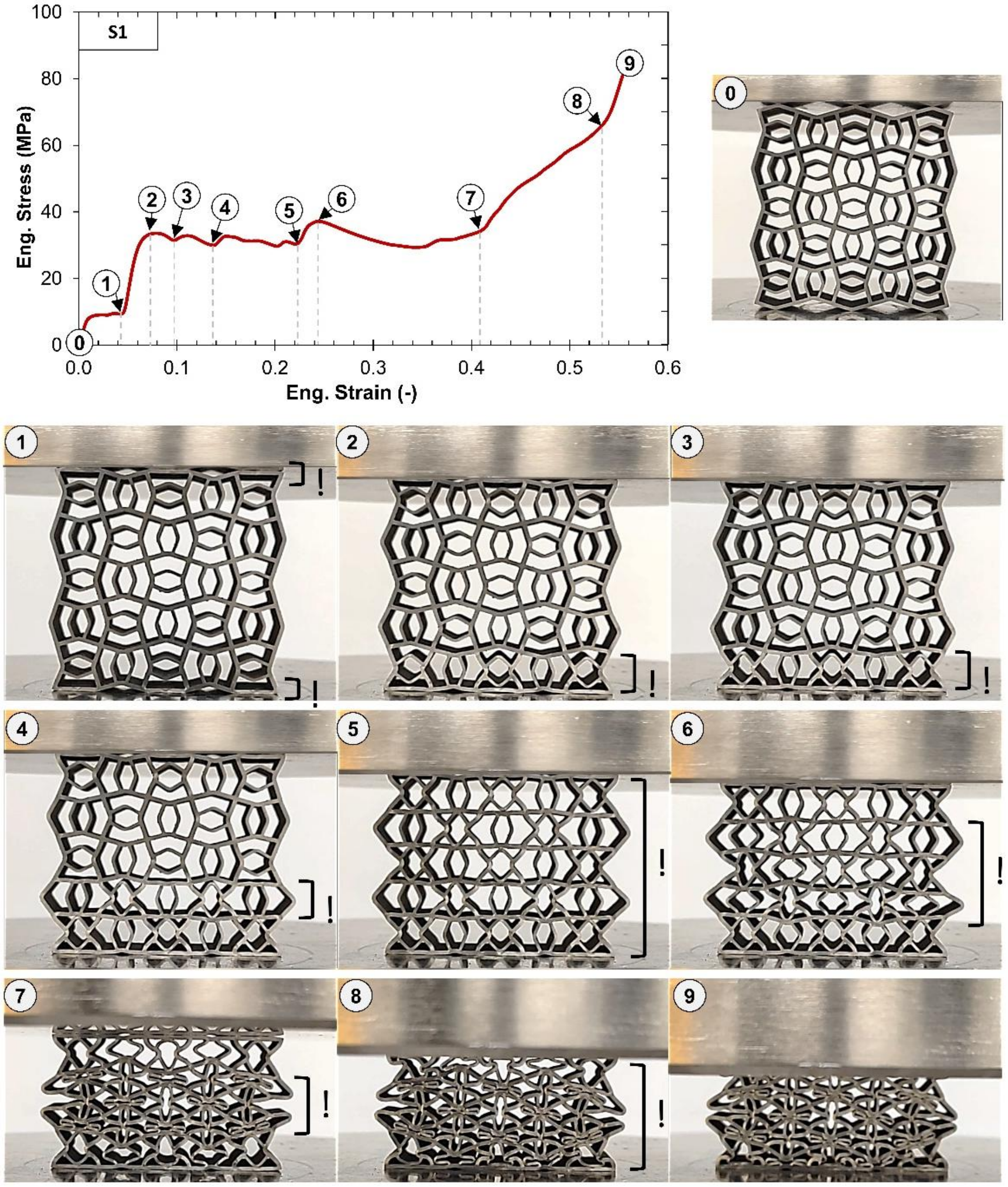

Figure D1. Detailed analysis of eng. stress-strain curve (experimental) of S1 structure

Supplement E

Video records of experimental compression test of designed structures (S0, S1, S2).

References

1. Li, Q. M., Magkiriadis, I. & Harrigan, J. J. Compressive strain at the onset of densification of cellular solids. *Journal of Cellular Plastics* **42**, 371–392 (2006).
2. Shinde, M. *et al.* Towards an Ideal Energy Absorber: Relating Failure Mechanisms and Energy Absorption Metrics in Additively Manufactured AlSi10Mg Cellular Structures under Quasistatic Compression. *Journal of Manufacturing and Materials Processing* **6**, (2022).
3. Gohar, S., Hussain, G., Ilyas, M. & Ali, A. Performance of 3D printed topologically optimized novel auxetic structures under compressive loading: experimental and FE analyses. *Journal of Materials Research and Technology* **15**, 394–408 (2021).
